# The Native *Wolbachia* Symbionts Limit Transmission of Dengue Virus in *Aedes albopictus*

**DOI:** 10.1371/journal.pntd.0001989

**Published:** 2012-12-27

**Authors:** Laurence Mousson, Karima Zouache, Camilo Arias-Goeta, Vincent Raquin, Patrick Mavingui, Anna-Bella Failloux

**Affiliations:** 1 Department of Virology, Arboviruses and Insect Vectors, Institut Pasteur, Paris, France; 2 UMR CNRS 5557, USC INRA 1193, VetAgro Sup, Ecologie Microbienne, Université de Lyon, Villeurbanne, France; Monash University, Australia

## Abstract

**Background:**

The chikungunya (CHIK) outbreak that struck La Reunion Island in 2005 was preceded by few human cases of Dengue (DEN), but which surprisingly did not lead to an epidemic as might have been expected in a non-immune population. Both arboviral diseases are transmitted to humans by two main mosquito species, *Aedes aegypti* and *Aedes albopictus*. In the absence of the former, *Ae. albopictus* was the only species responsible for viral transmission on La Reunion Island. This mosquito is naturally super-infected with two *Wolbachia* strains, *w*AlbA and *w*AlbB. While *Wolbachia* does not affect replication of CHIK virus (CHIKV) in *Ae. albopictus*, a similar effect was not observed with DEN virus (DENV).

**Methods/Principal Findings:**

To understand the weak vectorial status of *Ae. albopictus* towards DENV, we used experimental oral infections of mosquitoes from La Reunion Island to characterize the impact of *Wolbachia* on DENV infection. Viral loads and *Wolbachia* densities were measured by quantitative PCR in different organs of *Ae. albopictus* where DENV replication takes place after ingestion. We found that: (i) *Wolbachia* does not affect viral replication, (ii) *Wolbachia* restricts viral density in salivary glands, and (iii) *Wolbachia* limits transmission of DENV, as infectious viral particles were only detected in the saliva of *Wolbachia*-uninfected *Ae. albopictus*, 14 days after the infectious blood-meal.

**Conclusions:**

We show that *Wolbachia* does not affect the replication of DENV in *Ae. albopictus*. However, *Wolbachia* is able to reduce viral infection of salivary glands and limit transmission, suggesting a role of *Wolbachia* in naturally restricting the transmission of DENV in *Ae. albopictus* from La Reunion Island. The extension of this conclusion to other *Ae. albopictus* populations should be investigated.

## Author Summary

*Aedes albopictus* is an invasive species that is expanding its natural range of geographic distribution. While it was previously considered a secondary vector of different arboviruses, this mosquito species is involved in the most recent outbreaks of chikungunya but contributes weakly to dengue outbreaks. *Ae. albopictus* naturally carries two strains of the bacterium *Wolbachia*, *w*AlbA and *w*AlbB. Present in 20% of insect species, *Wolbachia* is an obligate intracellular symbiont mainly transmitted through females. When inoculated into some mosquito hosts, *Wolbachia* is able to shorten the adult life span and to block arbovirus transmission. We have previously shown that *Wolbachia* is not capable of limiting chikungunya replication in the mosquito vector. In this study, we show that the native *Wolbachia* is able to limit dengue transmission by restricting the delivery of infectious viral particles from the mosquito saliva when biting. Therefore, our results might explain the low vector competence of *Ae. albopictus* for dengue, and thus its weak contribution as an epidemic dengue vector.

## Introduction

Dengue is the most important arboviral disease in tropical and subtropical areas where 2.5 billion people are at risk of infection. The four dengue virus serotypes (DENV-1, -2, -3, -4) are transmitted by *Aedes* genus mosquitoes, mainly *Aedes aegypti* and *Aedes albopictus*. *Ae. aegypti* presents feeding preferences restricted to humans and is considered the main epidemic vector of DENV. *Ae. albopictus* is rather regarded as a secondary vector, although it has been implicated in several dengue outbreaks, and its geographic expansion is proceeding through tropical and temperate regions [1]. Currently, no specific antiviral therapy or vaccines are available against dengue. Thus, vector population control remains the principal tool for dengue prevention. Nevertheless, classical control methods such as the use of insecticides are failing to prevent a global increase in the incidence of dengue, and new approaches against this disease are needed.

*Wolbachia* can induce reproductive distortions in their hosts [2] increasing the reproductive success of infected females, thus enhancing their maternal transmission [3]. The ability of some *Wolbachia* strains to reduce the *Ae. aegypti* mosquito lifespan and to interfere with the replication of several pathogens, including arboviruses in this mosquito, has promoted this symbiotic bacterium as one of the most promising tools to limit virus transmission [4], [5]. To be transmitted successfully to the next host, DENV must replicate and disseminate in the mosquito's hemocoel before reaching the salivary glands. This period within the mosquito is called the extrinsic incubation period (EIP), and it determines how long a mosquito has to survive after having ingested the infectious blood-meal before becoming competent to transmit the virus. Only female mosquitoes that live longer than the EIP are of epidemiological interest. Therefore, a reduction in the number of old mosquitoes in a population may break disease transmission [6], [7].

*Wolbachia* strains can protect *Drosophila* flies from different RNA viruses resulting in infections showing lower titers and less associated pathologic effects [8], [9]. Infection of the naturally *Wolbachia*-free *Ae. aegypti* with different *Wolbachia* strains generates mosquitoes with increased resistance to viral infection and a shortened lifespan [10]–[12]. However, *Wolbachia*-mediated antiviral protection is not ubiquitous [13]. Natural *Wolbachia* strains that infect their native mosquito hosts have also been shown to induce resistance to viruses, although at a lower level than mosquito-transinfected strains [14].

*Wolbachia* is commonly found in mosquitoes including *Ae. albopictus*. This species is naturally super-infected with two *Wolbachia* strains, *w*AlbA and *w*AlbB [15], [16] and is able to transmit both DENV and chikungunya virus (CHIKV). On La Reunion Island, *Ae. albopictus* is the only species capable of sustaining an arboviral outbreak, because *Ae. aegypti* is scarcely present [17]. The former is a very efficient vector of a particular variant of CHIKV [18], [19] and, to a lesser extent, of DENV [1]. *Wolbachia* is widely found in natural populations of *Ae. albopictus* [20], [21] and is known to spread by inducing cytoplasmic incompatibility (CI) leading to successful increase of bacterial transmission through the female germline [22]. Although it is still unclear how *Wolbachia* interferes with arboviruses in the different tissues of the mosquito where they coexist, mechanisms such as stimulation of the host immune responses or competition for resources have been proposed [11], [12], [23]. *Wolbachia* is not able to limit DENV dissemination in *Ae. albopictus*, whereas a *Wolbachia*-mediated inhibition was observed in *Ae. aegypti* transfected with *w*AlbB [12]. It has been demonstrated that the *Wolbachia* density in somatic tissues of *Ae. albopictus*, such as midgut and salivary glands, is too low to induce resistance to DENV. Indeed, a strong negative linear correlation was observed between *Wolbachia* density and DENV load [24]. Nevertheless, when the *w*Mel strain of *Drosophila melanogaster* was introduced into *Ae. albopictus*, a complete blockade of DENV transmission was observed suggesting that in addition to the host background, the *Wolbachia* strain plays a key role in viral inhibition [25].

Our previous studies have shown that *Wolbachia* does not suppress CHIKV replication in *Ae. albopictus* [26]. Here, we examined whether *Wolbachia* is able to suppress DENV in *Ae. albopictus,* which is a poor DENV vector on La Reunion Island [27].

## Materials and Methods

### Ethics statement

The Institut Pasteur animal facility has received accreditation from the French Ministry of Agriculture to perform experiments on live mice [see permit numbers at http://webcampus.pasteur.fr/jcms/c_97619/agrements-des-animaleries] in compliance with the French and European regulations on care and protection of the Laboratory Animals. This study was approved by the relative IACUC at the Institut Pasteur.

### Mosquito rearing

Two mosquito strains were used in experiments: the F2 generation of the wild-type superinfected *Ae. albopictus* ALPROV (La Reunion Island) with *w*AlbA and *w*AlbB strains and the aposymbiotic line generated by antibiotic treatments of ALPROV (tetracycline for larval stage and rifampicin for adult stage) [26]. The ALPROV strain was generated from eggs collected in ovitraps placed in different houses in the “La Providence” neighborhood in the city of Saint-Denis. Obtained adults were maintained on a 10% sucrose at 28°C and 80% of relative humidity with a 16/8 light/dark cycle. Larvae were reared in pans with 200 larvae in 1 L of dechlorinated water, and fed with 1 yeast tablet every 2 days. The resulting adults were continuously provided with 10% sucrose. For routine colony maintenance, females were blood-fed three times a week on anesthetized mice.

### Experimental infections with DENV-2

The DENV-2 strain provided by Prof. Leon Rosen was isolated from a human serum collected in Bangkok (Thailand) in 1974. This virus had been passed in different mosquito species (*Toxorhynchites amboinensis*, *Ae. albopictus* and *Ae. aegypti*) by intrathoracic inoculation [28]. Viral production and titration are described in [29]. Blood-meals consisted of 1 mL of viral suspension in L-15 medium supplemented with 2% fetal bovine serum (FBS) and 2 mL of washed rabbit erythrocytes supplemented with ATP (5×10^−3^ M) as a phagostimulant. The infectious blood was transferred to a glass feeder maintained at 37°C and placed on top of plastic boxes containing 60 one-week-old mosquitoes that had been starved for 24 hours before the blood-meal. Mosquitoes from each box were allowed to feed for 20 min. Fully engorged females were transferred into cardboard containers and supplied with 10% sucrose at 28°C before being assayed.

### qRT-PCR and q-PCR assays

Individual mosquitoes and dissected organs (midguts, wings, and salivary glands) were used to extract total nucleic acids. At different days post-infection (pi), 5–9 mosquitoes were washed in 70% ethanol, and then rinsed twice in sterile PBS. The midgut, wings, and salivary glands were dissected in sterile PBS. Each organ was rinsed individually in PBS before being transferred separately into tubes containing 350 µL RA1 solution (NucleoSpin RNA II kit, Macherey-Nagel), then homogenized with the mixer Mill MM 300 (Qiagen). Needles used for dissection were decontaminated after each set of mosquitoes. Total RNA/DNA was extracted using the NucleoSpin RNA/DNA buffer set (Macherey-Nagel) coupled to the NucleoSpin RNA II kit. RNA was used to determine viral load by qRT-PCR, and DNA to measure *Wolbachia* (*w*AlbA and *w*AlbB) density and actin gene content by qPCR.

To measure the number of DENV-2 genome copies, the total RNA extracted was eluted into 40 µL RNAse-free water and 5 µL were used in a one-step RT-PCR reaction performed with a Power SYBR Green RNA-to-CT one step kit (Applied Biosystem) in a volume of 25 µL. The reaction contained 5 µL RNA template, 12.5 µL 2× Power SYBR Green I RT-PCR Mix, 0.625 µL sense primer (10 µM), 0.625 µL anti-sense primer (10 µM), 0.2 µL RT enzyme mix and 6.02 µL ddH_2_O. Primers were selected in the capsid structural protein coding region and were: sense D1-Lm/C/153/+ (GAG AAA CCG CGT GTC AAC TG) and anti-sense TS2-Lm/C/219/- (GGA AAC GAA GGA ATG CCA CC). The PCR program was: 48°C for 30 min, 95°C for 10 min; 40 cycles of 95°C for 15 s, and 60°C for 1 min; 90°C for 15 s with a final ramping of 19 min 59 sec. The size of the PCR product was 113 bp. A standard curve was generated using duplicates of 10^2^ to 10^8^ synthetic RNA transcripts per reaction. Quantification of viral RNA was achieved by comparing the threshold cycle (Ct) values of samples to those of standards according to the ΔC_t_ analysis. RNA viral copy number can give an overestimation of infectious viral particles and are more appropriate to describe viral dissemination than to estimate transmission probability.

To quantify *Wolbachia* (*w*AlbA and *w*AlbB) and *actin* genes, total DNA was extracted and used for q-PCR. The *Wolbachia* genome copy number was normalized using the nuclear *actin* gene. A plasmid (pQuantAlb), provided by Weill M [30] containing the three *w*AlbA, *w*AlbB *wsp* genes and the *Ae. albopictus actin* gene, was diluted from 10^2^ to 10^8^ copies/reaction and used to generate standard curves. Primers were: actAlb-dir (GCA AAC GTG GTA TCC TGA C) and actAlb-rev (GTC AGG AGA ACT GGG TGC T), QAdir1 (GGG TTG ATG TTG AAG GAG) and QArev2 (CAC CAG CTT TTA CTT GAC C), 183F (AAG GAA CCG AAG TTC ATG) and QBrev2 (AGT TGT GAG TAA AGT CCC), for *w*AlbA, *w*AlbB and *actin*, respectively. From 60 µL DNA solution extracted from one mosquito, 2 µL DNA was mixed with 0.3 µM of each primer and 12.5 µL FastStart Universal SYBR Green Master (Roche). PCR was run for 40 cycles (95°C for 10 min, 95°C for 15 sec, 60°C for 1 min). The mean number of *w*AlbA and *w*AlbB genomes was given per *actin* copy.

### Transmission assay

Mosquitoes exposed to a DENV-2 blood-meal were assayed for transmission at days 8 and 14 pi [31] by collecting saliva using the forced salivation technique [32]. Briefly, mosquitoes were anesthetized on ice to remove their legs and wings. Then the proboscis was inserted into a capillary tube containing 5 µL of fetal bovine serum. After 45 min, saliva was transferred into 45 µL of L15 medium.

### Forming assay on C6/36 cells

Saliva samples were titrated by fluorescent focus assay on C6/36 *Ae. albopictus* cell culture. Samples were serially diluted and inoculated into C6/36 cells in 96-well plates. After incubation at 28°C for 5 days, plates were stained using hyper-immune ascetic fluid specific to DENV-2 as the primary antibody and a goat anti-mouse conjugate as the second antibody.

### Fluorescence *in situ* hybridization (FISH)

Salivary glands were dissected from mosquitoes 14 days after exposure to an infectious blood-meal at a titer of 10^7^ FFU (fluorescent focus units)/mL. Preparations of samples were as described [30]. Briefly, organs were fixed for 20 min in freshly prepared 4% formaldehyde in PBS, and then washed once with PBS. Samples were incubated for 16 hours at 37°C in hybridization buffer [formamide 50%, SSC 5×, dextran sulfate 200 mg.l^−1^, poly(A) 250 mg.ml^−1^, salmon sperm DNA 250 mg.ml^−1^, tRNA 250 mg.ml^−1^, DTT 0.1 M, Denhartdt's solution 0.5×] containing 10 ng of two *Wolbachia* probes [30], [31] and two DENV specific probes [33]. Samples were washed twice in 1× SSC-10 mM DTT and twice in 0.5× SSC-10 mM DTT at 55°C for 15 min. Samples were then rinsed in PBS, mounted on a glass slide with glycerol and viewed under a confocal microscope (LSM510, Zeiss) at the Microscopy Centre of University Lyon I.

### Survival assay

1-week-old *Wolbachia*-infected and *Wolbachia-*uninfected females were fed on a DENV-2 blood-meal as described previously [29]. Engorged females were individually isolated in 50 mL tubes, maintained at 28°C and fed with 10% sucrose until death. Dead mosquitoes were scored daily to estimate the female life duration following exposure to an infectious blood-meal at a titer of 10^7^ FFU/mL.

### Statistical analysis

Statistical analyses were performed with Stata software (StataCorp LP, Texas, and USA). Means were compared using the Kruskal-Wallis test and the Khi2 test was used to compare rates.

## Results

### *Wolbachia* does not affect DENV-2 replication in *Ae. albopictus*

**Figure 1:**
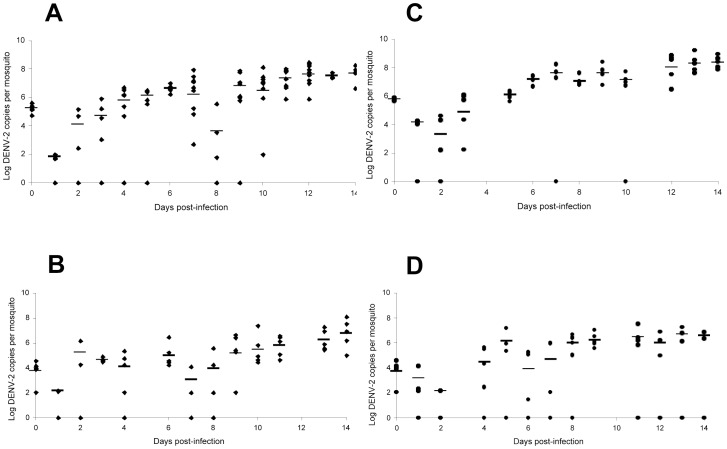
Viral dynamics in *Wolbachia*-infected and *Wolbachia*-uninfected *Ae. albopictus* after exposure to DENV-2. Batches of mosquitoes were exposed to an infectious blood-meal at two viral titers: 10^7^ FFU/mL (A, C) and 10^5^ FFU/mL (B, D). Every day, 4–9 mosquitoes were killed for RNA extraction and the number of DENV-2 genome copies was determined by qRT-PCR using primers targeting the C gene. Lines indicate the median. Significance was determined using the Mann-Whitney test (p<0.05).

**Figure 2:**
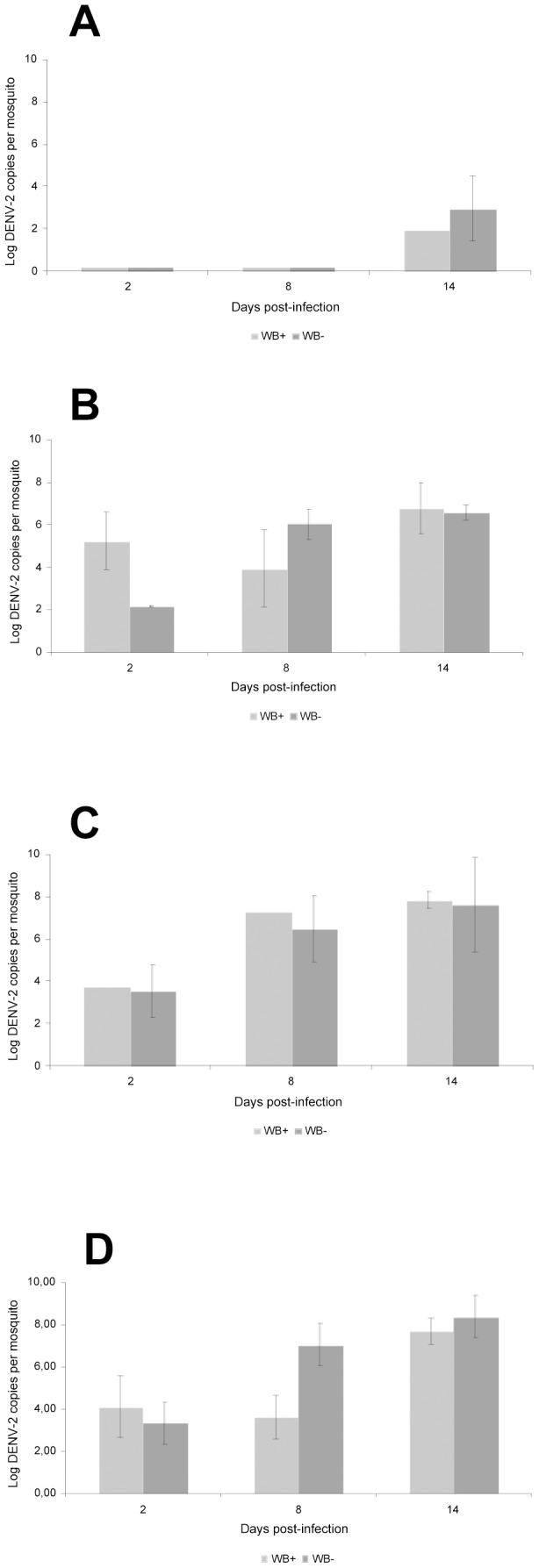
Viral loads in *Wolbachia*-infected and *Wolbachia*-uninfected *Ae. albopictus* after DENV-2 blood-meals provided at different titers. Mosquitoes were exposed to a DENV-2 blood-meal provided at four viral titers, 10^4^ (A), 10^5^ (B), 10^6^ (C) and 10^7^ (D) FFU/mL. At days 2, 8 and 14 pi, 4–9 mosquitoes were killed for RNA extraction and the number of DENV-2 genome copies was determined by qRT-PCR using primers targeting the C gene. Error bars indicate standard errors. Significance was determined using the Mann-Whitney test (p<0.05).

**Figure 3:**
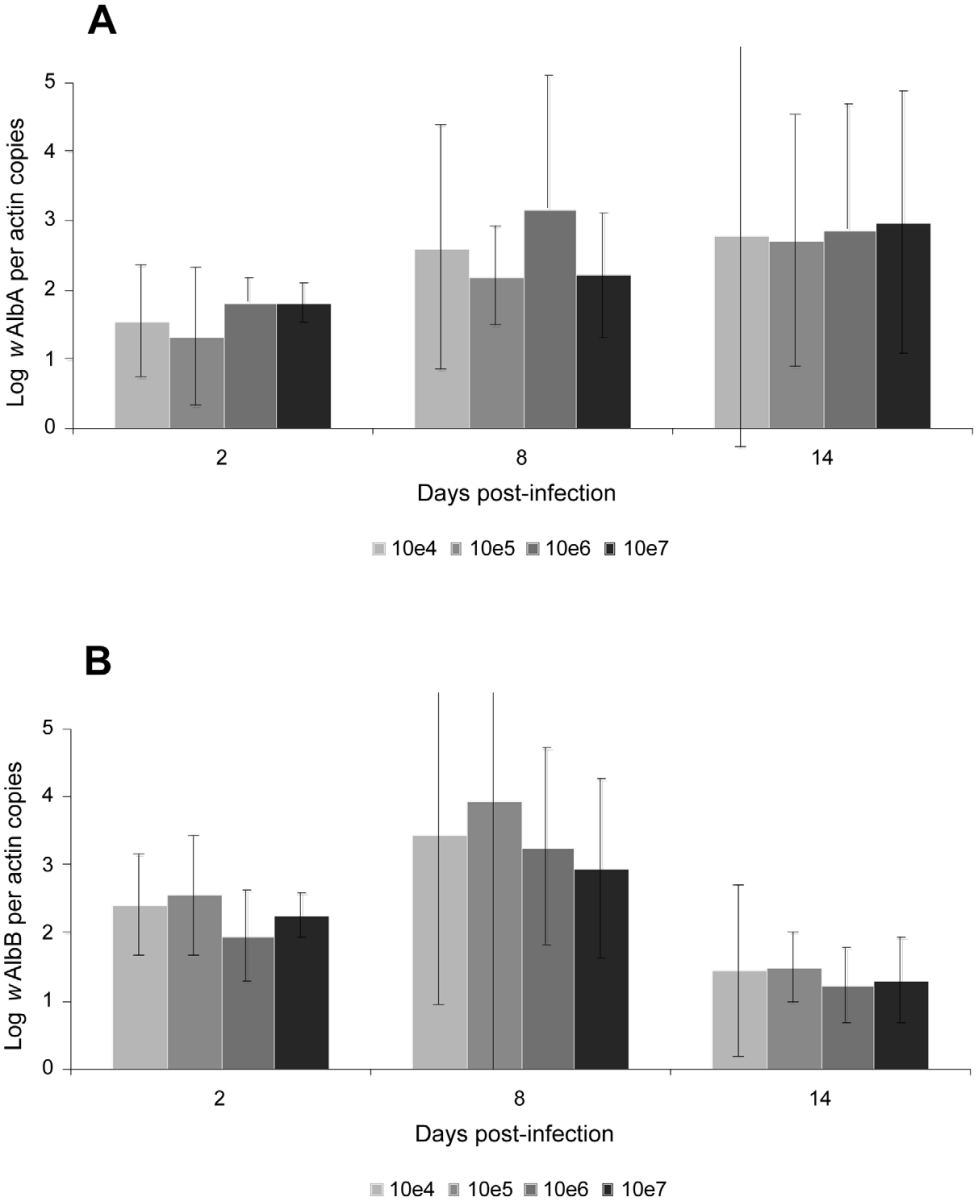
*Wolbachia* densities in *Ae. albopictus* after a DENV-2 blood-meal provided at different titers. At days 2, 8 and 14 pi, 4–9 mosquitoes were individually used for DNA extraction. q-PCR was conducted using primers targeting the *w*AlbA (A) and *w*AlbB (B) genes. The *Wolbachia* copy number was normalized with the *Ae. albopictus* actin gene. A plasmid (pQuantAlb) containing the three loci *w*AlbA, *w*AlbB, and the *Ae. albopictus* actin gene was serially diluted to build standard curves. Error bars represent standard errors.

To determine whether *Wolbachia* in *Ae. albopictus* affects DENV-2 replication, we fed *Wolbachia*-infected and *Wolbachia*-uninfected mosquitoes on a blood-meal proposed at two viral titers, 10^5^ and 10^7^ FFU/mL, and determined DENV-2 dynamics in mosquitoes. At a given viral titer of blood-meal, the number of DENV-2 genome copies in whole individuals was not significantly different between *Wolbachia*-infected and *Wolbachia*-uninfected strains (Mann-Whitney test: p>0.05) (Figure 1). Later, we compared DENV-2 loads in mosquitoes between *Wolbachia*-infected and *Wolbachia*-uninfected *Ae. albopictus* exposed to four viral titers, 10^4^, 10^5^, 10^6^, and 10^7^ FFU/mL. At days 2, 8, and 14 pi, the number of DENV-2 genome copies estimated by qRT-PCR was not significantly different between *Wolbachia*-infected and *Wolbachia*-uninfected mosquitoes at a given viral titer of blood-meal (Mann-Whitney test: p>0.05) (Figure 2). Concomitantly, at days 2, 8, and 14 pi, the loads of *w*AlbA (Figure 3A) and *w*AlbB (Figure 3B) remained roughly similar whatever the viral titer of blood-meal (Kruskall-Wallis test: p>0.05), albeit the *Wolbachia* densities were highly variable between mosquito individuals and day pi. Nevertheless, it was interesting to note that when a slight increase of *w*AlbA loads was observed between day 8 pi and day 14 pi, a concomitant decrease of *w*AlbB loads was detected. This result was not in line with previous findings with CHIKV [26].

### *Wolbachia* restricts densities of DENV-2 in *Ae. albopictus* salivary glands

**Figure 4:**
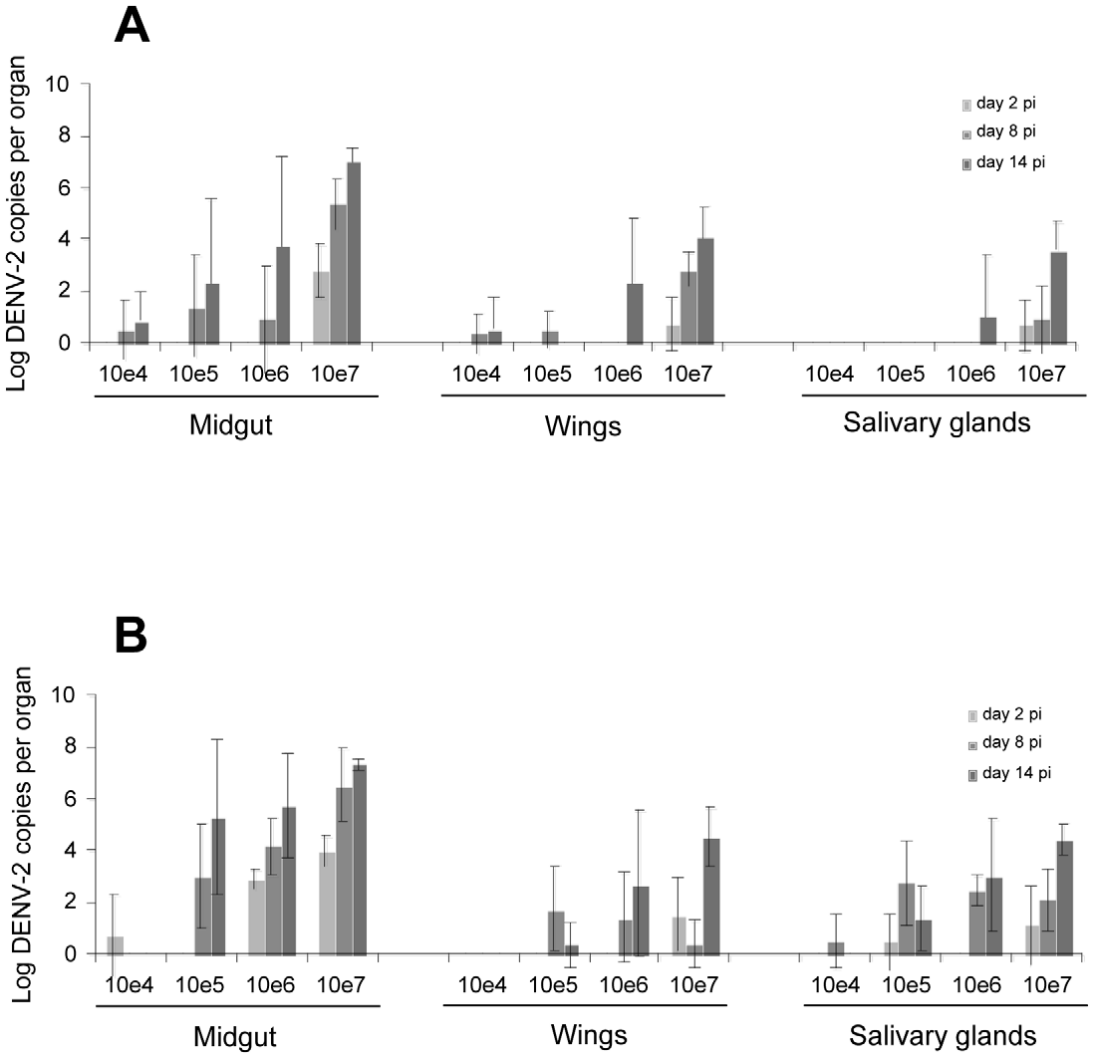
DENV-2 infection and dissemination in *Wolbachia*-infected and *Wolbachia*-uninfected *Ae. albopictus*. *Wolbachia*-infected (A) and *Wolbachia*-uninfected (B) mosquitoes were exposed to a DENV-2 blood-meal provided at 4 different titers, 10^4^, 10^5^, 10^6^, 10^7^ FFU/mL. At days 2, 8 and 14 pi, midguts, wings and salivary glands were collected, and the number of DENV-2 genome copies was determined by qRT-PCR using primers targeting the C gene. Error bars indicate standard errors.

**Figure 5:**
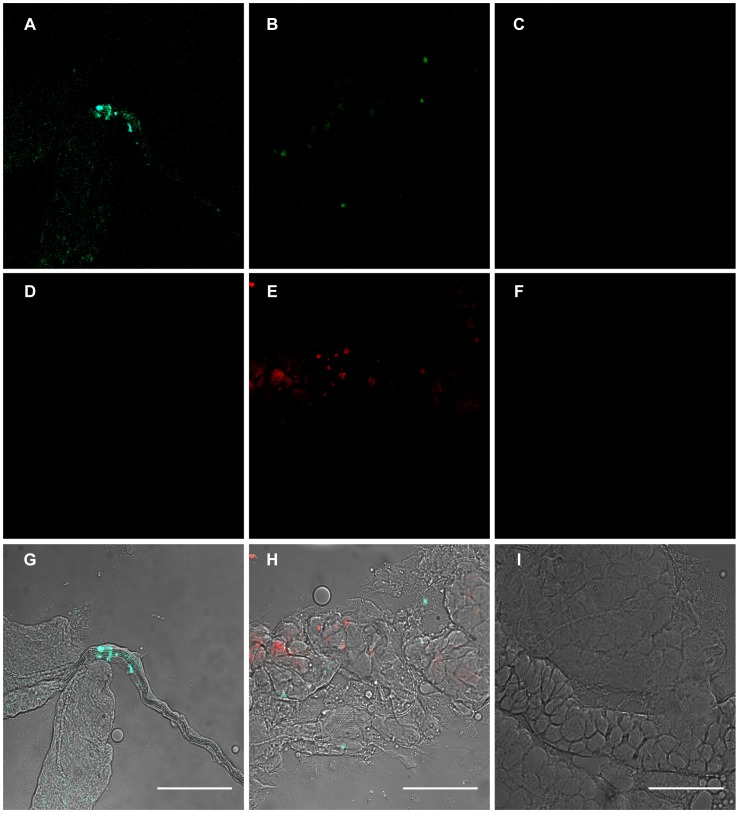
Localization of *Wolbachia* and DENV-2 in *Ae. albopictus.* At 14 days post-DENV-2 infection, salivary glands were dissected, fixed, and then incubated simultaneously with two *Wolbachia* probes and two DENV specific probes. In panels A, B, and C, DENV-2 (green) is labeled with FITC. In panels D, E, and F, *Wolbachia* (red) is stained with Rhodamine. In panels G, H, and I, the red and green channels are merged. A co-localization of *Wolbachia* and DENV-2 was detected in some cells (panel H). DENV-uninfected and *Wolbachia*-uninfected controls are presented in panel C and F, respectively. Scale bars: 50 µm.

Since the overall DENV-2 replication in *Ae. albopictus* did not differ between *Wolbachia*-infected and *Wolbachia*-uninfected mosquitoes, we compared the capacity of DENV to disseminate from the midgut to secondary organs by estimating viral loads in wings and salivary glands at days 2, 8, and 14 days after exposure of mosquitoes to blood-meals provided at four viral titers: 10^4^, 10^5^, 10^6^, and 10^7^ FFU/mL. The viral load in the midgut increased with the titer of the ingested blood-meal and the duration post-infection. In *Wolbachia*-infected mosquitoes, although virus dissemination to the wings was correlated with the blood-meal titer and day pi (Figure 4A). However, a strong reduction of viral load was observed in wings of *Wolbachia*-infected mosquitoes compared to *Wolbachia*-uninfected ones, suggesting that virions produced in the midgut epithelial cells were not massively released into the hemocoel. Thus, the occurrence of a midgut escape barrier in *Wolbachia*-infected *Ae. albopictus* can be suggested. Finally, salivary glands were only heavily infected at day 14 pi at the highest blood-meal titer assessed of 10^7^ FFU/mL. Failure of DENV-2 to infect salivary glands might be due to low viral loads detected in the hemolymph (e.g. wings). On the other hand, in *Wolbachia*-uninfected mosquitoes, profiles observed in the different organs were significantly different with those observed in *Wolbachia*-infected mosquitoes (Kruskall-Wallis test: p<0.05). Indeed, DENV-2 loads in the midgut started to increase intensively from a blood-meal at 10^5^ FFU/mL (Figure 4B). Dissemination from the midgut to wings and salivary glands remained relatively high at different titers in *Wolbachia*-uninfected individuals. Therefore, in *Wolbachia*-uninfected mosquitoes, DENV-2 did not seem to be restricted for dissemination to secondary organs suggesting the occurrence of a weak midgut escape barrier. Higher viral loads were found in *Wolbachia*-uninfected organs than in *Wolbachia*-infected ones, including salivary glands. Using specific FISH probes, we detected DENV-2 genomic RNA (green) (Figure 5, panels A, B, and C) and *Wolbachia* DNA (red) (Figure 5, panels D, E, and F). A co-localization of both *Wolbachia* and DENV-2 was detected in salivary glands (Figure 5, panel H). However, DENV-2 signals were relatively higher in *Wolbachia-*uninfected salivary glands (Figure 5, panel A) compared to a few dots in *Wolbachia-*infected ones (Figure 5, panel B), which is in agreement with PCR quantification data. As expected, no hybridization signal was observed in the salivary glands of DENV-uninfected and *Wolbachia*-uninfected individuals used as negative controls (Figure 5, panels C and F respectively).

### *Wolbachia* limits DENV-2 transmission in *Ae. albopictus*

30–40 mosquitoes that survived until day 14 pi were examined for transmission. DENV-2 particles were only detected in the saliva of *Wolbachia*-uninfected *Ae. albopictus*, 14 days after an infectious blood-meal at a titer of 10^7^ FFU/mL; only 12.5% of mosquitoes (among 32 tested) were able to deliver viral particles (mean ± standard deviation: 18±28) (data not shown). This suggests that a salivary gland escape barrier preventing the release of virus from salivary glands operates in *Wolbachia*-uninfected *Ae. albopictus*. In addition, *Wolbachia*'s effect depends on the viral titer of the blood-meal.

### *Wolbachia* does not increase the longevity of DENV-2 infected *Ae. albopictus*

**Figure 6:**
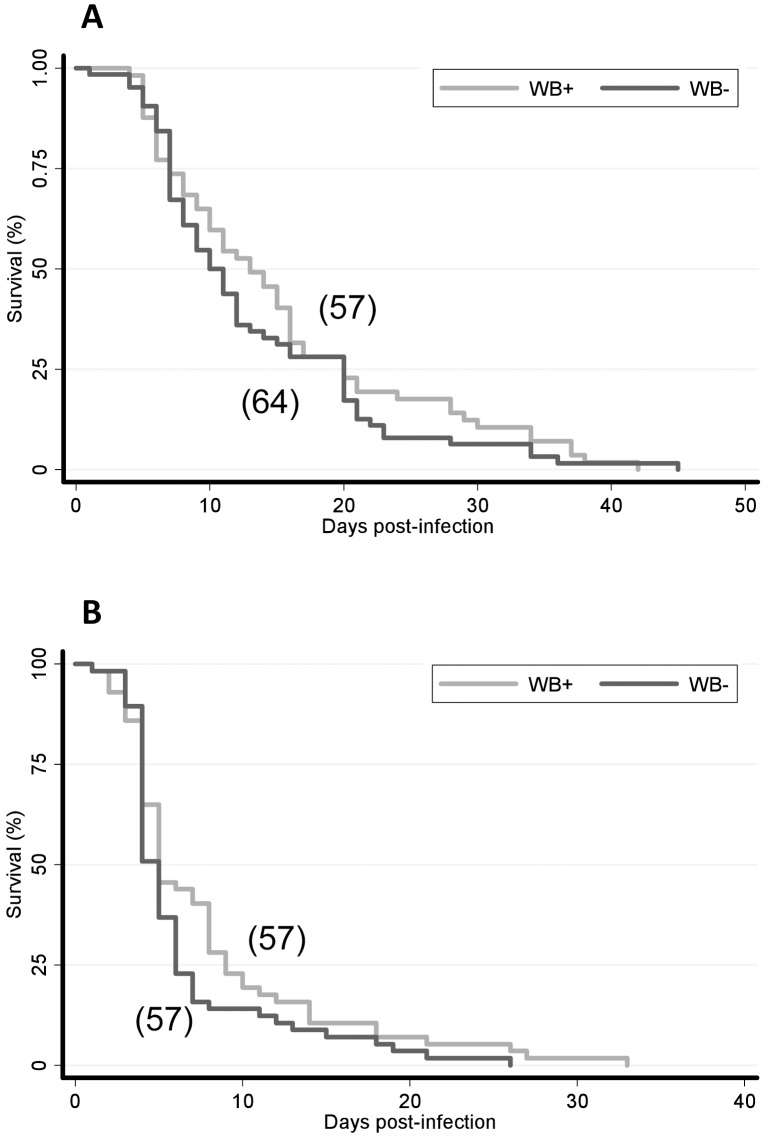
Survival of *Wolbachia*-infected and *Wolbachia*-uninfected *Ae. albopictus* after infection with DENV-2. Mosquitoes were fed on a DENV-2 blood-meal at a titer of 10^7^ FFU/mL and maintained at 28°C. Two replicates (A, B) are shown. The survival curves were not significantly different between *Wolbachia*-infected and *Wolbachia*-uninfected mosquitoes fed with DENV-2 (logrank test, p>0.05). In brackets, the number of females is given.

Removing *Wolbachia* did not affect *Ae. albopictus* survival when non-infected with DENV (data not shown). Then, to determine whether removing *Wolbachia* has an effect on DENV-2 infected mosquitoes, we compared the survival of *Wolbachia*-infected and *Wolbachia*-uninfected mosquitoes after infection with DENV-2 in two trials (Figure 6). Neither mosquito strains showed any difference in survival curves (logrank test: p>0.05). These results indicated that *Wolbachia* did not affect the longevity of *Ae. albopictus* when mosquitoes are infected with DENV-2.

## Discussion

Considering the overlapping distribution of *Wolbachia* and DENV in non-reproductive tissues of mosquitoes, such as midgut and salivary glands, we conducted experiments to characterize the role of *Wolbachia* in DENV-2 transmission by *Ae. albopictus*. Our results show that *Wolbachia* naturally infecting *Ae. albopictus* limits DENV-2 dissemination and the infection of salivary glands in the ALPROV strain, suggesting that *Ae. albopictus* is a poor epidemic vector of DENV in La Reunion Island [1].

*Wolbachia* is present in the midgut and salivary glands, two organs crucial for viral transmission [34]. DENV penetrates into the midgut epithelial cells of mosquitoes following an infectious blood-meal. After replication and release of virions from the midgut, secondary organs such as salivary glands are subsequently infected. Infectious viral particles are then released from the salivary glands through saliva when the mosquito bites [35]. Replication of DENV reaches a maximum in the mosquito at day 7–8 pi, as was observed for the principal DENV vector *Ae. aegypti* [31]. *Wolbachia* densities in *Ae. albopictus* were not affected by the blood-meal titer; when increasing the blood-meal titer, *w*AlbA and *w*AlbB remained stable. This was consistently observed at different days pi (2, 8, and 14). Nevertheless, high levels of variation of *Wolbachia* densities were observed between individuals of the recently laboratory-established population of *Ae. albopictus* from La Reunion, as has been reported with mosquitoes from Thailand [20]. When examining the midgut infection, we found that the viral loads in the midgut increased when the titer of the ingested blood-meal increased, as observed for other arboviruses [36], [37]. In addition, virus dissemination to wings and salivary glands was correlated with the virus titer of blood-meals. Interestingly, we found that *Wolbachia* might have an inhibitory effect on DENV-2 dissemination. Indeed, the dissemination of infectious virions from the midgut to secondary organs, such as wings and salivary glands, was affected, suggesting the presence of a midgut escape barrier. Nevertheless, such a barrier could be overwhelmed when the blood-meal is provided at a titer higher than 10^6^–10^7^ FFU/mL. Later, the release of virus from salivary glands into the saliva of *Wolbachia*-infected mosquitoes at day 14 pi was completely inhibited suggesting the existence of a salivary gland escape barrier, possibly exerted by *Wolbachia* [38]. Probably, at higher titers of blood-meals, this barrier may be less efficient. Thereby, a speculative suggestion can be made: if *Ae. albopictus* naturally carrying *Wolbachia* receives a high titer of blood-meal (>10^7^ FFU/mL), the inhibition exerted by *Wolbachia* on DENV can be overcome. Thus viral transmission from a viremic patient to a mosquito vector is more likely to occur when high titers of blood-meals are provided. This can happen with the invasion and expansion of a new viral clade, resulting in high viremias in humans. Consequently, this leads to an enhanced mosquito transmission for the newly introduced viral lineage [39], [40]. These invasive lineages causing higher viral titers are usually associated with more severe symptoms in humans [41]. Conversely, in *Wolbachia*-uninfected mosquitoes, midgut infection was intensive and dissemination to wings and salivary glands was not strongly limited. Virus was only detected in saliva at day 14 pi in 12.5% of *Wolbachia*-uninfected mosquitoes. Contrary to *Wolbachia*-infected *Ae. albopictus*, *Wolbachia*-uninfected mosquitoes do not show the inhibition caused by a midgut escape barrier as well as a salivary gland escape barrier. Therefore, removing *Wolbachia* from *Ae. albopictus* also removes the inhibition exerted by *Wolbachia* on DENV-2 in *Ae. albopictus.* Low transmission to saliva might explain the low vector competence of *Ae. albopictus* for DENV. However, increasing the incubation period may favor transmission; in *Ae. albopictus* from Lebanon, virus was only detected at day 21 pi with 38% of mosquitoes delivering 174±455 (mean ± standard deviation) viral particles, whereas no viral particles were detected in the saliva at day 14 pi (Haddad N et al., unpublished data). Thus, the extended extrinsic incubation period of DENV in *Ae. albopictus* which requires that females live longer to be capable of transmitting, may contribute to reduce the potential for transmission of *Ae. albopictus* populations and weaken the role of the species as epidemic dengue vector.

The inhibitory effect of *Wolbachia* on different pathogens including DENV was demonstrated in the vector *Ae. aegypti* [11], [12]. Similar interference was not found in *Ae. albopictus* naturally infected by *Wolbachia*. Both strains, *w*AlbA and *w*AlbB, are benign to *Ae. albopictus* [42] and contrary to reports on *Drosophila* [8], [9]. It is not clear whether *Wolbachia* is able to remain a benign symbiont simply by maintaining a very low replication rate independent of the host cell or whether the bacterium actively coordinates its replication with the host through specific immune mechanisms. Immune responses to *Wolbachia* infection have only been observed in hosts artificially infected with new strains of *Wolbachia* [11], [43], [44]. However, responses might not be solely be due to the *Wolbachia* infection but could reflect a consequence of the new host-symbiont combination such as elevated, unnatural *Wolbachia* densities [45]. High densities of *Wolbachia* can provide high levels of protection whereas low densities provide limited protection [13], [24]. Interference could also result from a direct competition between DENV and *Wolbachia* for the same resources or from an indirect disturbance by *Wolbachia* on the cellular environment required by DENV for its replication. Interestingly, replication of CHIKV in *Ae. albopictus* was concomitant with a decrease of *Wolbachia* densities [26], in contrast with the DENV replication pattern observed in the present study. The intensive replication of CHIKV in *Ae. albopictus* reaching up to 10^9^ viral RNA from day 3 pi [32] induced negative effects on mosquito survival [37]. Contrary to DENV, CHIKV has seemingly developed a subtle balance between intense replication and adverse effects in mosquitoes, though limited enough to maintain a high transmission capacity.

To conclude, our study showed that native *Wolbachia* was able to limit transmission of DENV in *Ae. albopictus*. Presumably, introducing an additional infection to the preexisting infection [46] or a new *Wolbachia* type in *Wolbachia*-free *Ae. albopictus* [25], [47] might lead to a blockade of viral replication and dissemination making *Ae. albopictus* refractory to DENV.

## References

[1] Lambrechts L, Scott TW, Gubler DJ (2010) Consequences of the expanding global distribution of *Aedes albopictus* for dengue virus transmission. PLoS Negl Trop Dis 4: e646. 20520794 10.1371/journal.pntd.0000646 PMC2876112

[2] Landmann F, Orsi GA, Loppin B, Sullivan W (2009) *Wolbachia*-mediated cytoplasmic incompatibility is associated with impaired histone deposition in the male pronucleus. PLoS Pathog 5: e1000343. 19300496 10.1371/journal.ppat.1000343 PMC2652114

[3] Werren JH, Baldo L, Clark ME (2008) *Wolbachia*: master manipulators of invertebrate biology. Nat Rev Microbiol 6: 741–751. 18794912 10.1038/nrmicro1969

[4] Walker T, Johnson PH, Moreira LA, Iturbe-Ormaetxe I, Frentiu FD, et al (2011) The *w*Mel *Wolbachia* strain blocks dengue and invades caged *Aedes aegypti* populations. Nature 476: 450–453. 21866159 10.1038/nature10355

[5] Hoffmann AA, Montgomery BL, Popovici J, Iturbe-Ormaetxe I, Johnson PH, et al (2011) Successful establishment of *Wolbachia* in *Aedes* populations to suppress dengue transmission. Nature 476: 454–457. 21866160 10.1038/nature10356

[6] Brownstein JS, Hett E, O'Neill SL (2003) The potential of virulent *Wolbachia* to modulate disease transmission by insects. J Invertebr Pathol 84: 24–29. 13678709 10.1016/s0022-2011(03)00082-x

[7] Sinkins SP, O'Neill SL (2000) *Wolbachia* as a vehicle to modify insect populations. Boca Raton, FLUSA: CRC Press.

[8] Hedges LM, Brownlie JC, O'Neill SL, Johnson KN (2008) *Wolbachia* and virus protection in insects. Science 322: 702. 18974344 10.1126/science.1162418

[9] Teixera L, Ferreira A, Ashburner M (2008) The bacterial symbiont *Wolbachia* induces resistance to RNA viral infections in *Drosophila melanogaster* . PLoS Biol 6: e2. 10.1371/journal.pbio.1000002 PMC2605931 19222304

[10] McMeniman CJ, Lane RV, Cass BN, Fong AW, Sidhu M, et al (2009) Stable introduction of a life-shortening *Wolbachia* infection into the mosquito *Aedes aegypti* . Science 323: 141–144. 19119237 10.1126/science.1165326

[11] Moreira LA, Iturbe-Ormaetxe I, Jeffery JA, Lu G, Pyke AT, et al (2009) A *Wolbachia* symbiont in *Aedes aegypti* limits infection with dengue, Chikungunya, and Plasmodium. Cell 139: 1268–1278. 20064373 10.1016/j.cell.2009.11.042

[12] Bian G, Xu Y, Lu P, Xie Y, Xi Z (2010) The endosymbiotic bacterium *Wolbachia* induces resistance to dengue virus in *Aedes aegypti* . PLoS Pathog 6: e1000833. 20368968 10.1371/journal.ppat.1000833 PMC2848556

[13] Osborne SE, Leong YS, O'Neill SL, Johnson KN (2009) Variation in antiviral protection mediated by different *Wolbachia* strains in *Drosophila simulans* . PLoS Pathog 5: e1000656. 19911047 10.1371/journal.ppat.1000656 PMC2768908

[14] Glaser RL, Meola MA (2010) The native *Wolbachia* endosymbionts of *Drosophila melanogaster* and *Culex quinquefasciatus* increase host resistance to West Nile virus infection. PLoS ONE 5: e11977. 20700535 10.1371/journal.pone.0011977 PMC2916829

[15] Sinkins SP, Braig HR, O'Neill SL (1995) *Wolbachia* superinfections and the expression of cytoplasmic incompatibility. Proc Biol Sci 261: 325–330. 8587875 10.1098/rspb.1995.0154

[16] Zhou W, Rousset F, O'Neill SL (1998) Phylogeny and PCR based classification of *Wolbachia* strains using *wsp* gene sequences. Proc Biol Sci 265: 509–515. 9569669 10.1098/rspb.1998.0324 PMC1688917

[17] Salvan M, Mouchet J (1994) *Aedes albopictus* et *Aedes aegypti* à l'Ile de La Réunion. Ann Soc Belge Med Trop 74: 323–326. 7726666

[18] Vazeille M, Moutailler S, Coudrier D, Rousseaux C, Khun H, et al (2007) Two Chikungunya isolates from the outbreak of La Reunion (Indian Ocean) exhibit different patterns of infection in the mosquito, *Aedes albopictus* . PLoS ONE 2: e1168. 18000540 10.1371/journal.pone.0001168 PMC2064959

[19] Tsetsarkin KA, Vanlandingham DL, McGee CE, Higgs S (2007) A single mutation in chikungunya virus affects vector specificity and epidemic potential. PLoS Pathog 3: e201. 18069894 10.1371/journal.ppat.0030201 PMC2134949

[20] Ahantarig A, Trinachartvanit W, Kittayapong P (2008) Relative *Wolbachia* density of field-collected *Aedes albopictus* mosquitoes in Thailand. J Vector Ecol 33: 173–177. 18697321 10.3376/1081-1710(2008)33[173:rwdofa]2.0.co;2

[21] Zouache K, Raharimalala FN, Raquin V, Tran-Van V, Raveloson LH, et al (2011) Bacterial diversity of field-caught mosquitoes, *Aedes albopictus* and *Aedes aegypti*, from different geographic regions of Madagascar. FEMS Microbiol Ecol 75: 377–389. 21175696 10.1111/j.1574-6941.2010.01012.x

[22] Kambhampati S, Rai KS, Burgun SJ (1993) Unidirectional cytoplasmic incompatibility in the mosquito, *Aedes albopictus* . Evolution 47: 673–677. 28568710 10.1111/j.1558-5646.1993.tb02121.x

[23] Frentiu FD, Robinson J, Young PR, McGraw EA, O'Neill SL (2010) *Wolbachia*-mediated resistance to dengue virus infection and death at the cellular level. PLoS ONE 5: e13398. 20976219 10.1371/journal.pone.0013398 PMC2955527

[24] Lu P, Bian G, Pan X, Xi Z (2012) *Wolbachia* induces density-dependent inhibition to dengue virus in mosquito cells. PLoS Negl Trop Dis 6: e1754. 22848774 10.1371/journal.pntd.0001754 PMC3404113

[25] Blagrove MSC, Arias-Goeta C, Failloux AB, Sinkins SP (2012) The *Wolbachia* strain *w*Mel induces cytoplasmic incompatibility and blocks dengue transmission in *Aedes albopictus* . Proc Natl Acad Sci USA 109: 255–260. 22123944 10.1073/pnas.1112021108 PMC3252941

[26] Mousson L, Martin E, Zouache K, Madec Y, Mavingui P, et al (2010) *Wolbachia* modulates Chikungunya replication in *Aedes albopictus* . Mol Ecol 19: 1953–1964. 20345686 10.1111/j.1365-294X.2010.04606.x

[27] Paupy C, Girod R, Salvan M, Rodhain F, Failloux AB (2001) Population structure of *Aedes albopictus* from La Réunion Island (Indian Ocean) with respect to susceptibility to a dengue virus. Heredity 87: 273–283. 11737274 10.1046/j.1365-2540.2001.00866.x

[28] Rosen L, Gubler D (1974) The use of mosquitoes to detect and propagate dengue viruses. Am J Trop Med Hyg 23: 1153–1160. 4429185 10.4269/ajtmh.1974.23.1153

[29] Vazeille-Falcoz M, Mousson L, Rodhain F, Chungue E, Failloux AB (1999) Variation in oral susceptibility to dengue type 2 virus of populations of *Aedes aegypti* from the islands of Tahiti and Moorea, French Polynesia. Am J Trop Med Hyg 60: 292–299. 10072154 10.4269/ajtmh.1999.60.292

[30] Tortosa P, Courtiol A, Moutailler S, Failloux AB, Weill M (2008) Chikungunya-*Wolbachia* interplay in *Aedes albopictus* . Insect Mol Biol 17: 677–684. 19133077 10.1111/j.1365-2583.2008.00842.x

[31] Salazar MI, Richardson JH, Sánchez-Vargas I, Olson KE, Beaty BJ (2007) Dengue virus type 2: replication and tropisms in orally infected *Aedes aegypti* mosquitoes. BMC Microbiol 7: 9. 17263893 10.1186/1471-2180-7-9 PMC1797809

[32] Dubrulle M, Mousson L, Moutailler S, Vazeille M, Failloux AB (2009) Chikungunya virus and *Aedes* mosquitoes: saliva is infectious as soon as two days after oral infection. PLoS ONE 4: e5895. 19521520 10.1371/journal.pone.0005895 PMC2690823

[33] Raquin V, Wannagat M, Zouache K, Legras-Lachuer C, Moro CV, et al (2012) Detection of dengue group viruses by fluorescence *in situ* hybridization. Parasit Vectors 5: 243. 23110979 10.1186/1756-3305-5-243 PMC3507901

[34] Zouache K, Voronin D, Tran-Van V, Mousson L, Failloux AB, et al (2009) Persistent *Wolbachia* and cultivable bacteria infection in the reproductive and somatic tissues of the mosquito vector *Aedes albopictus* . PLoS ONE 4: e6388. 19633721 10.1371/journal.pone.0006388 PMC2712238

[35] Kramer LD, Ebel GD (2003) Dynamics of flavivirus infection in mosquitoes. Adv Virus Res 60: 187–232. 14689695 10.1016/s0065-3527(03)60006-0

[36] Focks DA, Daniels E, Haile DG, Keesling JE (1995) A simulation model of the epidemiology of urban dengue fever: literature analysis, model development, preliminary validation, and samples of simulation results. Am J Trop Med Hyg 53: 489–506. 7485707 10.4269/ajtmh.1995.53.489

[37] Martin E, Moutailler S, Madec Y (2010) Differential responses of the mosquito *Aedes albopictus* from the Indian Ocean region to two chikungunya isolates. BMC Ecol 10: 8. 20226023 10.1186/1472-6785-10-8 PMC2847964

[38] Black WC 4th, Bennett KE, Gorrochótegui-Escalante N, Barillas-Mury CV, Fernández-Salas I, et al (2002) Flavivirus susceptibility in *Aedes aegypti* . Arch Med Res 33: 379–388. 12234528 10.1016/s0188-4409(02)00373-9

[39] Hanley KA, Nelson JT, Schirtzinger EE, Whitehead SS, Hanson CT (2008) Superior infectivity for mosquito vectors contributes to competitive displacement among strains of dengue virus. BMC Ecol 8: 1. 18269771 10.1186/1472-6785-8-1 PMC2263032

[40] Lambrechts L, Fansiri T, Pongsiri A, Thaisomboonsuk B, Klungthong C, et al (2012) Dengue-1 virus clade replacement in Thailand associated with enhanced mosquito transmission. J Virol 86: 1853–1861. 22130539 10.1128/JVI.06458-11 PMC3264336

[41] Vaughn DW, Green S, Kalayanarooj S, Innis BL, Nimmannitya S, et al (2000) Dengue viremia titer, antibody response pattern, and virus serotype correlate with disease severity. J Infect Dis 181: 2–9. 10608744 10.1086/315215

[42] Dobson SL, Fox CW, Jiggins FM (2002) The effect of *Wolbachia*-induced cytoplasmic incompatibility on host population size in natural and manipulated systems. Proc Biol Sci 269: 437–45. 11886634 10.1098/rspb.2001.1876 PMC1690924

[43] Hussain M, Frentiu FD, Moreira LA, O'Neill SL, Asgari S (2011) *Wolbachia* uses host microRNAs to manipulate host gene expression and facilitate colonization of the dengue vector *Aedes aegypti* . Proc Natl Acad Sci USA 108: 9250–9255. 21576469 10.1073/pnas.1105469108 PMC3107320

[44] Pan X, Zhou G, Wu J, Bian G, Lu P, et al (2012) *Wolbachia* induces reactive oxygen species (ROS)-dependent activation of the Toll pathway to control dengue virus in the mosquito *Aedes aegypti* . Proc Natl Acad Sci USA 109: E23–31. 22123956 10.1073/pnas.1116932108 PMC3252928

[45] McGraw EA, Merritt DJ, Droller JN, O'Neill SL (2002) *Wolbachia* density and virulence attenuation after transfer into a novel host. Proc Natl Acad Sci USA 99: 2918–2923. 11880639 10.1073/pnas.052466499 PMC122448

[46] Fu Y, Gavotte L, Mercer DR, Dobson SL (2010) Artificial triple *Wolbachia* infection in *Aedes albopictus* yields a new pattern of unidirectional cytoplasmic incompatibility. Appl Environ Microbiol 76: 5887–5891. 20601501 10.1128/AEM.00218-10 PMC2935066

[47] Sun LV, Riegler M, O'Neill SL (2003) Development of a physical and genetic map of the virulent *Wolbachia* strain *w*MelPop. J Bacteriol 185: 7077–7084. 14645266 10.1128/JB.185.24.7077-7084.2003 PMC296261

